# The Comparison of PCR Kits for the Detection of Erythrocytic Parasites on Filter Paper

**DOI:** 10.1155/2022/5715436

**Published:** 2022-08-13

**Authors:** Zhi-yong Tao, Pei-yi Zhang, Lu Zhang, Chun-cao Li, Rui Hu, Han-wu Zhu, Bei Zhou, Kai Wu, Ling-xu Li, Da-wei Yao, Yu-jie Cao, Dao-jin Wang, Chen-chen Zheng, Run-qi Fang, Xiu-min Hua, Yi-xuan Ni, Xiao-xia Jin, Hui Xia, Qiang Fang

**Affiliations:** ^1^Department of Microbiology and Parasitology, Bengbu Medical College, 2600 Donghai Avenue, Bengbu 233030, China; ^2^Anhui Key Laboratory of Infection and Immunology, Bengbu Medical College, 2600 Donghai Avenue, Bengbu 233030, China; ^3^Chenzhou Center for Disease Control and Prevention, Chenzhou 423000, Hunan, China; ^4^College of Veterinary Medicine, Hunan Agricultural University, Changsha 410128, Hunan, China; ^5^Wuhan Center for Disease Control and Prevention, Wuhan 430022, Hubei, China; ^6^College of Veterinary Medicine, Nanjing Agricultural University, No. 1 Weigang Road, Nanjing 210095, China

## Abstract

Dried blood spot (DBS) based PCR was considered an inexpensive and feasible method for detecting pathogens in the blood. The DBS carrier filter paper and PCR kits are crucial for accurate diagnosis. We evaluated 4 types of filter papers and 20 PCR kits for DBS samples. The PCR detecting *Plasmodium* results showed that the minimum detection limit of the 4 filter papers was 1 × 10^2^ parasites/*μ*L, and the positive rates of 20 PCR kits ranged from 0% to 100%. PCR results were satisfactory for detecting *Plasmodium falciparum* (*P. falciparum*) and *Plasmodium. vivax* (*P. vivax*) in archived DBS samples and *Babesia gibsoni* (*B. gibsoni*) in fresh pet DBS samples. Our results provided a useful reference for the detection of blood pathogens with DBS samples and direct PCR, especially for screening the cost-efficacy combination of filter paper and PCR kit in resource-limited areas.

## 1. Introduction

*Plasmodium* [1–3] and *Babesia* [4–8] are two major intraerythrocytic parasites causing human and animal infection, namely malaria and babesiosis. Both are life-threatening. Fast and accurate diagnosis of these diseases is crucial for the treatment of patients and to contain the spread of these vector-borne diseases. Microscopy is the most common method for laboratory diagnosis of blood parasitic infection, but low parasitemia infection may lead to misdiagnosis. Sometimes, the identification of species of *Plasmodium* or *Babesia* may not be possible due to morphological similarity. More importantly, *Babesia* is easily confused with early-stage *Plasmodium* [3, 6, 9, 10] and can lead to unsuccessful treatment with antimalarial drugs. PCR-based molecular diagnostic approaches are important supplementary methods for accurate diagnosis [11–15], but these methods are usually not feasible in resource-limited areas due to equipment requirements and cost.

Since the 1960s, dried blood spot (DBS) samples have been used in the clinical diagnostic analysis for blood testing, mainly for the screening of neonatal metabolic disorders and genetic abnormalities [16–20]. Many DBS-based experimental methods have been successfully developed, such as analysis methods for nucleic acids, lipids, and other substances. Qualitative and quantitative detection based on DBS PCR has been widely used in the screening of infectious diseases such as HIV, HBV, CMV, and so on. It is also of great importance in the detection of parasitic infections in the blood [21–24], especially for *Plasmodium* infection [1, 9, 25, 26].

Collecting whole blood on filter paper as dried blood spot samples was optimal for the storage and transportation of blood samples from the field site to the laboratory for centralized testing [17]. As a sampling tool for PCR molecular diagnosis and surveillance, the quality of filter paper is particularly important. Among many types of filter paper, Whatman 903 filter paper has FDA approval and is widely used. Currently, 903 filter paper is provided as a relatively expensive ready-to-use sampling card. Whatman CF12 filter paper is a cheaper form of square sheet filter paper and is currently listed as one item of 903 Proteinsaver cards by its new manufacturer Cytiva. CF 12 filter paper is globally available and marketed as 903 filter paper in China. However, a comparison of physical properties and molecular detection performance of CF12 and other filter papers was not usually done.

At present, in order to facilitate the rapid control of many infectious diseases, there is a need for efficient and convenient nucleic acid collection and detection methods in the field. Existing PCR test requires a long detection time, and samples such as throat swabs and blood need to be extracted for nucleic acid before testing, which increases the overall diagnosis time. Moreover, the cost of nucleic acid extraction and detection is high, and some underdeveloped regions do not have proper nucleic acid extraction equipment and laboratory settings. Therefore, there was an urgent need for the development of rapid detection methods.

Direct PCR is one of the most valued rapid molecular detection techniques. Before 1993, direct PCR was used in the field of microbiology, for which the sample processing is very simple [19]. For example, colony PCR is a typical rapid method for screening colonies. Most direct PCR is based on the use of a genetically modified DNA polymerase, which makes it possible for bypassing nucleic acid extraction before PCR amplification, saving experimental materials and time. Moreover, it reduces the possibility of human error and sample cross-contamination [1]. The requirements for polymerase are high, not only in the tolerance to interferential components but also in the compatibility with the buffer of PCR reaction.

There are potent PCR inhibitors in crude samples such as blood, which can cause false negative PCR results [27]. Several major inhibitory components in blood have been characterized, that is, hemoglobin, immunoglobulin *G*, and lactoferrin. The mechanism is related to the inactivation or inhibition of *Taq* DNA polymerase, which may reduce the efficiency and usability of direct PCR based on filter paper blood samples [28, 29].

We purchased four types of filter papers commercially available in mainland China and evaluated them in terms of weight, thickness, blood absorption performance, positive rates, and price. A total of 20 commercially available PCR kits, mainly for direct PCR of blood samples, were selected and tested using DBS samples. The aim of this research is to evaluate the cost-effectiveness of DNA polymerases that were suitable for molecular diagnosis of blood infection in DBS samples.

## 2. Materials and Methods

### 2.1. Filter Papers

Whatman CF12 (GE healthcare, UK), Advantec 545 (Advantec Group, Tokyo, Japan), and Jesiman Filter Paper (Jesiman New Material Co. Ltd., Wuhan, China) were purchased from the Taobao platform in China (https://taobao.com), and Gel Blot Paper was purchased from Sangon Biotech Co. Ltd. (Shanghai, China). Supplementary Table S1 details this information.

### 2.2. The Preparation of DBS Filter Paper Samples Containing *Plasmodium* parasites

**Figure 1:**
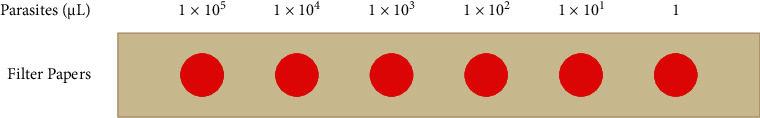
The scheme of the preparation of dried blood spot samples by tenfold serial diluting *Plasmodium yoelii*-infected blood.

Collect EDTA-K2 anticoagulated blood from mice infected with *P. yoelii*. Adjust the parasitemia to 1 × 10^5^ parasites/*μ*L, and 1, 10^1^, 10^2^, 10^3^, 10^4^, and 10^5^ parasites/µL blood samples were obtained by tenfold serial dilution with EDTA-K2 anticoagulated whole blood. Blood was absorbed by four types of filter papers to prepare DBS samples, each spot contains 50 µL of blood. The blood spot preparation schematic diagram is shown in Figure 1.

*Plasmodium falciparum* 3D7 (*P. falciparum*) dried blood spots: four levels of parasite density blood samples (1 and 10^1^ to 10^3^ parasites/µL) were obtained by tenfold serial dilution and absorbed on CF12 filter paper.

The DBS samples were placed on a clean bench and dried for 48 h. The filter papers were stored in a sealed plastic bag with silica gel desiccant.

### 2.3. Self-Made Blood Collection Card

The self-made blood collection cards are made with CF12 filter paper, consisting of upper and lower cover and enclosed sampling filter (see Supplementary Figure S1). It was placed upright to dry the blood samples after collection and to protect the DBS samples during transportation. Each DBS sampling card was individually packaged with silica gel desiccant in a plastic bag and stored at room temperature.

### 2.4. DBS Samples of Malaria-Infected Blood

Our laboratory has established a repository of malaria samples obtained over decades of research. Preserved DBS samples of *Plasmodium vivax* (*P. vivax*) infected blood were collected in Wuhe County, China, between 2009 and 2014, stored at −20°C. The DBS samples of *P. falciparum*-infected blood were collected from imported cases mostly from African countries, between 2010 and 2013, and stored at room temperature.

### 2.5. Animal Blood Samples

Self-made blood collection cards were sent to the pet hospitals located in three cities: Bengbu, Changsha, and Nanjing. DBS samples were collected from dogs. After complete drying, DBS samples were sent back to our lab for detection of *Babesia* infection.

### 2.6. Pretreatment of Blood Spots before PCR Reaction

Two pieces of dried blood spots (equivalent to 3–5 µL of whole blood) were punched with a 1.5 mm puncher and placed in one PCR reaction tube. To remove the hemoglobin in the sample and dust floating on the surface of the blood spots, 70 µL of sterilized ultrapure water was added to each tube, and the reaction tubes were incubated at 50°C for 5 min, 21°C for 15 s, 50°C for 1.5 min, and 21°C for 15 s. The supernatant in the tubes was then aspirated and discarded.

### 2.7. Primers Used for PCR Detection of Pathogens

The target of nested PCR for *Plasmodium* was 18S rRNA. Genus-specific primers rPLU6 and rPLU5 were used for the first round of amplification, and the amplified products were used for the second round of amplification, in which the primers used were species-specific for *P. falciparum* and *P. vivax*. The target gene for the detection of *Babesia gibsoni* was 18S rRNA. The sequences of all primers used in this research are shown in Supplementary Table S2.

### 2.8. The Information of 20 Commercial DNA Polymerases

All 20 DNA polymerases were purchased from Labgic Technology Co. Ltd. (Hefei, China), and the purchasing information is shown in Supplementary Table S3.

### 2.9. PCR Conditions

For each PCR, all reagent components were added according to their product instructions. For different PCR, the detailed programs were listed in Supplementary Tables S4–S6. The annealing temperatures were set according to the Tm value of each primer pair, and the extension times are set according to the expected fragment length and the enzyme elongation speed. All PCR reactions were carried out on an S1000 Thermal Cycler (Bio-Rad, USA).

## 3. Results

### 3.1. The Characteristics of Four Types of Filter Papers

**Figure 2:**
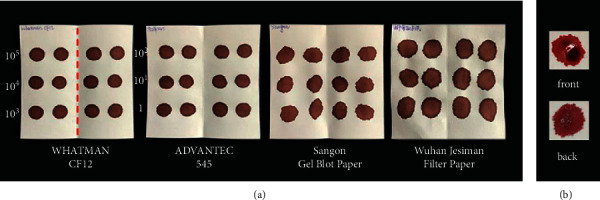
Dried blood spots formed by four filter papers after absorbing serial diluted *Plasmodium*-infected blood: (a) the shape of dried blood spots formed on four types of filter papers and (b) the leakage of blood during the formation of blood spots.

We compared the general properties of the four types of filter papers available from the mainland China market, including thickness, weight, and blood absorption ability. The results showed that there were differences in the absorption time and the diameter of the blood spots when using healthy human blood and laboratory mice blood. For the absorption time of 50 *μ*L whole blood by different filter papers, CF12 and 545 filter papers only take 3–5 seconds, which was significantly faster than the other two filter papers. CF12 and 545 filter papers formed more regular blood spots and were less prone to leakage (see Table 1). The Sangon Gel Blot paper could not completely absorb 50 *μ*L of blood in a short time, and the blood sample was seen to spread unevenly in the filter paper, while no similar diffusion and leakage occurred in the other three filter papers (see Figure 2).

### 3.2. Detection Limits and Positive Rates of Direct PCR on Four Types of Filter Papers

A high-fidelity polymerase (Tks Gflex, TAKARA R060Q) based PCR was used to determine the detection limit for *Plasmodium* in the dried blood spots of four filter papers. The detection target was a 134 bp fragment of *P. yoelii* 17XNL 18srRNA gene. The results showed that the minimum detection limit of all four filter papers was 1 × 10^2^ parasites/*μ*L.

For comparison of the efficiency of different filter papers for DBS-based PCR, we purchased 20 commercial DNA polymerases for PCR detection of erythrocytic parasites in the DBS sample. The *Plasmodium* genus-specific primer rPLU6/5 was used to amplify a 1,003 bp fragment of *P. yoelii* in the DBS sample. The results showed positive rates of four filter papers that ranged from 56.7% to 63.3% (see Table 2), and the chi-square test showed no significant difference between the four filter papers (*p* > 0.05).

### 3.3. The Results of 20 DNA Polymerases for Detection of *Plasmodium* Genes in DBS

For comparing the performance of 20 polymerases, serial diluted *P. yoelii* 17XNL DBS samples on four filter papers were tested. The results showed that the positive rates ranged from 0% to 100% (see Table 2). Five PCR kits can detect all *Plasmodium* samples with different parasite densities on four types of filter papers.

### 3.4. Cost-Effectiveness Analysis of 20 DNA Polymerases

In order to screen cost-effective DNA polymerases, both unit price and positive rate need to be considered. As well known, the less template DNA in samples, the more difficult to amplify, the cost-effective criteria were established according to the difficulty of amplification: 1 point for positive of 10^3^ parasites, 10 points for 10^2^ parasites, and 100 points for 10^1^ parasites (see Table 3). Eight DNA polymerases with positive rates from 80% to 100% were selected for the subsequent experiments.

### 3.5. Performance Comparison of DNA Polymerases for Detection of *P. falciparum*

Blood samples of cultured *P. falciparum* were tenfold serial diluted (1–10^3^ parasites/µL) by normal mice blood and DBS samples were prepared using CF12 filter paper. Eight selected DNA polymerases were tested for detection of *P. falciparum* using nested PCR techniques. The first-round primers rPLU5/6 were genus-specific, and the second-round primers rFAL1/2 were *P. falciparum*-specific. One microliter of first-round PCR product was used as the template DNA in the second-round PCR. The results showed that the following five DNA polymerases: Beyotime HemoTaq, NEB Hemo Klen *Taq*, TAKARA MightyAmp, TOYOBO KOD FX Neo, and Sangon Direct PCR Kit, were better for *P. falciparum*, with all positive rates at 100% (detailed amplification results are listed in Supplementary Table S7). These five DNA polymerases were selected for subsequent detection of clinical samples.

### 3.6. Direct PCR Results of Human Malaria Parasites in DBS Sample

Forty-one positive DBS samples of human malaria were retrieved from our laboratory archive and were analyzed by direct PCR using five cost-effective polymerases. The original filter papers used for preserving blood samples were not uniform: some of them were obvious thinner or thicker; some were prepared with an FTA card. Also, the density of parasites in each DBS sample was unclear. These samples consisted of 21 *P. falciparum* and 20 *P. vivax*. The results showed that the following three polymerases can detect both malaria parasites at 100% positivity rates: TAKARA MightyAmp, TOYOBO KOD FX Neo, and Sangon Direct PCR Kit. The regions from which these samples were taken and the results of the amplification of the five DNA polymerases are shown in Supplementary Table S8.

### 3.7. DBS-Sample-Based Direct PCR for Detection of *Babesia* in Dog Blood

In order to verify whether DBS direct PCR is suitable for detecting *Babesia* in dog blood, self-made CF12 blood collection cards were used to collect dog blood in three regional veterinary hospitals. DBS samples were sent back to our laboratory for the batch test. After receiving the samples, the direct PCR test was conducted by using only the MigthyAMP kit. The results showed that two positive samples were detected from Bengbu dog blood DBS samples. Sequencing of PCR product and Giemsa's stain confirmed that the two dogs were infected with *Babesia gibsoni*. The detailed results are shown in Supplementary Table S9.

## 4. Discussion

Due to limited resources in many tropical countries, it may not be possible to meet the basic storage requirements for blood samples for laboratory diagnostics. Many studies have shown that a variety of substances were stable in DBS and could be preserved for longer periods than traditional methods. DBS was considered an alternative to blood samples for the detection of antibodies or nucleic acid in resource-limited areas and in populations where venous blood collection, preservation, and transportation were difficult. However, differences in the nature of the filter papers could influence the analytic results, and the compatibility with key reagents and their uneven performance can also cause variable results of a DBS-based assay.

In this study, four commercially available filter papers were compared in terms of their performance in absorbing blood, price, and suitability for nucleic acid detection using 20 DNA polymerases. The Whatman CF12 and Advantec 545 filter papers exhibited excellent blood absorption properties and were more convenient for preparation but were relatively expensive. The comparison of nucleic acid detection performance showed that there was no significant difference in the positivity and detection limits between the four filter papers. And the results from the archived DBS samples made with a variety of filter papers for *P. falciparum* also showed that DBS samples made with much thinner filter paper can be effectively detected by direct PCR. The results of this experiment suggest that common laboratory filter paper could also provide nucleic acid detection support when there was no high-quality filter paper available.

The nucleic acids in DBS samples can be extracted by commercial kits with higher purity, but a relatively larger volume of eluent is needed, and the final concentration of DNA may be quite low. In this experiment, the amplification was carried out by using two pieces of punched filter paper placed directly in the PCR tube during the reaction. The punched DBS sample was treated with a mild and simple wash procedure, which can remove partial inhibitors in the blood and retain enough nucleic acids for successful detection in the sample, which helps improve the results, reduce cost, and save time.

It is known that PCR inhibitors are present in the blood, and requirements for reagents used in direct PCR of blood samples were high, especially polymerases. In this experiment, we compared the performance and cost-effectiveness of 20 commercially available DNA polymerases for DBS direct PCR. This technique does not need DNA extraction, simplifies the sample processing steps, and reduces the chance of possible contamination in the extraction step. Our results showed that most of the DNA polymerases can detect blood parasites in filter paper samples with high levels of parasite density (10^3^ parasites/µL), and some of them reach their minimum detection limits as the density of parasite decreases. Among all polymerases tested, five polymerases, Beyotime Hemo*Taq*, NEB Hemo Klen *Taq*, TAKARA MightyAmp, TOYOBO KOD FX Neo, and Sangon Direct PCR Kit, gave better results and were more resistant to inhibitors in the blood. However, some polymerases may not be suitable for DBS PCR. Furthermore, the experimental results of direct PCR on archived human malaria DBS samples showed that all five DNA polymerases had high detection rates, and the three DNA polymerases, TAKARA MightyAmp, TOYOBO KOD FX Neo, and Sangon Direct PCR Kit, had 100% positive rates for both human malaria tests.

In order to perform large-scale screening, the cost per test must be taken into account. By scoring the PCR results to compare the cost-efficiency of direct PCR reagents, the method used in this study can be used as a useful reference for regions with limited resources to evaluate kits that were available locally and to facilitate large-scale screening of pathogens. In the reaction system for PCR, we followed the product instructions without modification. In fact, after optimization of direct PCR conditions for each primer and template, the performance of polymerases may be improved to some extent.

This study suggests that a simple PCR nucleic acid detection technique based on filter paper and direct PCR kits with high sensitivity is suitable for the detection of pathogen genes within the blood, especially in resource-limited epidemic areas, and provides an economical and convenient option for large-scale screening of human and animal infectious disease by collecting filter paper blood samples in field sites and transporting them back to laboratories that are equipped to perform bulk testing.

## Figures and Tables

**Table 1:** Characteristics of four types of filter papers.

Filter papers	Whatman CF12	Advantec 545	Sangon Gel Blot paper	Wuhan Jesiman Filter Paper
Weight (g/m^2^)	182–187	177–182	184–187	110–115
Thickness (mm)	0.47–0.51	0.42–0.44	0.28–0.30	0.23–0.25
Human blood absorption time (s/50 *μ*l)	5	4	553–1072	35–68
Human blood spot diameter (mm)	11–12	11–13	10–15	15–18
Mice blood absorption time (s/50 *μ*l)	4	3	577–668	19–21
Mice blood spot diameter (mm)	12–13	13	12–15	17
Regularity of blood spot	Yes	Yes	No	No
Blood leakage (50 *μ*l)	No	No	Yes	Yes
Price (USD/m^2^)^*∗*^	39.66	29.05	23.56	17.44

^*∗*^The price was calculated based on the exchange rate: 1 CNY = 0.159 USD.

**Table 2:** DBS PCR results of 20 DNA polymerases.

DNA polymerases		Whatman CF12 (parasites/µL)			Advantec 545 (parasites/µL)			Gel Blot paper (parasites/µL)			Jesiman Filter Faper (parasites/µL)			Positive rates of 20 polymerases
		10^1^	10^2^	10^3^	10^1^	10^2^	10^3^	10^1^	10^2^	10^3^	10^1^	10^2^	10^3^	
Beyotime	Hemo*Taq*	＋	＋	＋	−	＋	＋	＋	＋	＋	−	＋	＋	83.30% (10/12)
	*Taq*	−	＋	＋	−	＋	＋	−	−	＋	−	−	＋	50.00% (6/12)
	Hemo*Taq* HF	−	−	−	−	−	−	−	−	−	−	−	＋	8.30% (1/12)
	BeyoFusion	−	−	−	−	−	−	−	−	−	−	−	−	0.00% (0/12)

Sangon biotech	Direct PCR kit	＋	＋	＋	＋	＋	＋	＋	＋	＋	＋	＋	＋	100.00% (12/12)
	*Taq* Plus	−	−	−	−	−	−	−	−	−	−	−	−	0.00% (0/12)

TAKARA	Tks Gflex	＋	＋	＋	−	＋	＋	＋	＋	＋	＋	＋	＋	91.70% (11/12)
	*Taq*	＋	＋	＋	−	＋	＋	−	＋	＋	＋	＋	＋	83.30% (10/12)
	MightyAmp	＋	＋	＋	＋	＋	＋	＋	＋	＋	＋	＋	＋	100.00% (12/12)

TOYOBO	r*Taq*	−	−	＋	−	−	＋	−	−	＋	−	＋	＋	41.70% (5/12)
	KOD Plus Neo	−	＋	＋	−	＋	＋	−	＋	＋	＋	＋	＋	75.00% (9/12)
	KOD FX Neo	＋	＋	＋	＋	＋	＋	＋	＋	＋	＋	＋	＋	100.00% (12/12)

TIANGEN	Mouse Tissue Direct PCR kit	−	＋	＋	＋	＋	＋	−	＋	＋	＋	＋	＋	83.30% (10/12)
	Ultra HiFidelity	＋	＋	＋	＋	＋	＋	＋	＋	＋	＋	＋	＋	100.00% (12/12)
FOREVERSTAR	Blood direct PCR kit	−	−	−	−	−	−	−	−	−	−	−	−	0.00% (0/12)

Thermo Fisher	Phire Hot Start II	−	＋	＋	−	＋	＋	−	−	＋	−	＋	＋	58.30% (7/12)
	Platinum direct PCR kit	−	＋	＋	−	＋	＋	−	＋	＋	−	−	＋	58.30% (7/12)

NEB	Hemo klen *Taq*	＋	＋	＋	＋	＋	＋	＋	＋	＋	＋	＋	＋	100.00% (12/12)
	Vent	−	−	−	−	−	−	−	−	−	−	−	−	0.00% (0/12)
	Q5	＋	＋	＋	＋	＋	＋	−	＋	＋	−	＋	＋	83.30% (10/12)

Positive rates of 4 filter papers		45.00% (9/20)	70.00% (14/20)	75.00% (15/20)	35.00% (7/20)	70.00% (14/20)	75.00% (15/20)	35.00% (7/20)	60.00% (12/20)	75.00% (15/20)	45.00% (9/20)	65.00% (13/20)	80.00% (16/20)	
				63.30% (38/60)			60.00% (36/60)			56.70% (34/60)			63.30% (38/60)			

**Table 3:** Cost-effectiveness comparison of 20 DNA polymerases.

DNA polymerases	Item no.	Price (USD)	Tests	Price/test (USD)	Positive rate (*n*/12) (%)	Scores^*∗*^	Scores (price/test)
NEB Hemo klen *Taq*	M0332S	186.55	200	0.93	100.00	444	476.00
TAKARA MightyAmp	R071Q	40.95	40	1.02	100.00	444	433.69
Ultra HiFidelity PCR kit	KP203	97.28	80	1.22	100.00	444	365.14
TOYOBO KOD FX Neo	KFX-201S	31.38	20	1.57	100.00	444	282.98
Sangon Direct PCR kit	B639289-0050	82.37	50	1.65	100.00	444	269.51
TAKARA Tks Gflex	R060Q	75.47	80	0.94	100.00	344	364.65
TAKARA *Taq*	R001 A	18.83	200	0.09	83.30	244	2,591.88
Beyotime Hemo*Taq*	D7241S	39.70	200	0.20	83.30	244	1,229.35
Mouse Tissue Direct PCR kit	KG205	62.76	50	1.26	83.30	244	194.39
NEB Q5	M0491S	137.92	100	1.38	83.30	244	176.92
TOYOBO KOD Plus Neo	KOD-401S	23.54	20	1.18	75.00	144	122.37
Phire Hot Start II	F122S	220.76	200	1.10	58.30	34	30.80
Platinum Direct PCR kit	A44647100	136.97	100	1.37	58.30	34	24.82
Beyotime Beyotime *Taq*	D7205	4.39	160	0.03	50.00	24	874.08
TOYOBO r*Taq*	TAP-211	23.54	100	0.24	41.70	14	59.49
Beyotime Hemo*Taq* HF	D7243S	47.85	200	0.24	8.30	1	4.18
Beyotime BeyoFusion	D7220	16.95	80	0.21	0.00	0	0.00
Sangon *Taq* Plus	B500013-0100	3.45	20	0.17	0.00	0	0.00
StarLighter Blood Direct PCR	FS-P6001	18.83	500	0.04	0.00	0	0.00
NEB Vent	M0254 V	61.03	200	0.31	0.00	0	0.00

^*∗*^Scores were calculated based on the PCR results of 20 polymerases: 1 point for positive of 10^3^ parasites, 10 points for 10^2^ parasites, and 100 points for 10^1^ parasites.

## Data Availability

The data that support the findings of this study are available in the manuscript as well as in the supplementary materials of this article.

## Supplementary Materials

Supplementary Materials The supplementary materials contain nine tables showing filter papers and DNA polymerases purchasing details; sample collecting information; and PCR primers, conditions, and results. In addition, Supplementary Figure S1 shows the self-made blood collection card based on CF12 filter paper.
