# Supplementary material for: The Comparison of PCR Kits for the Detection of Erythrocytic Parasites on Filter Paper
Source: J Trop Med. 2022 Aug 13;2022:5715436. doi: 10.1155/2022/5715436 (PMC9392634; doi:10.1155/2022/5715436)
Supplement: Supplementary Materials — The supplementary materials contain nine tables showing filter papers and DNA polymerases purchasing details; sample collecting information; and PCR primers, conditions, and results. In addition, Supplementary Figure S1 shows the self-made blood collection card based on CF12 filter paper. [file 5715436.f1.doc]

**Table S1. Four filter papers purchasing details**

| **Filter Papers** | **CF12** | **545** | **Gel Blot Paper** | **Jesiman Filter Paper** |
| --- | --- | --- | --- | --- |
| **Manufacturer** | Whatman | Advantec | Sangon | Jesiman |
| **Item no.** | 10538018 | 545 | F513323-0001 | N/A |
| **Size (mm×mm)** | 580×580 | 350×540 | 150×150 | 300×300 |
| **Price (USD)** | 13.34 | 5.49 | 0.53 | 1.57 |

**
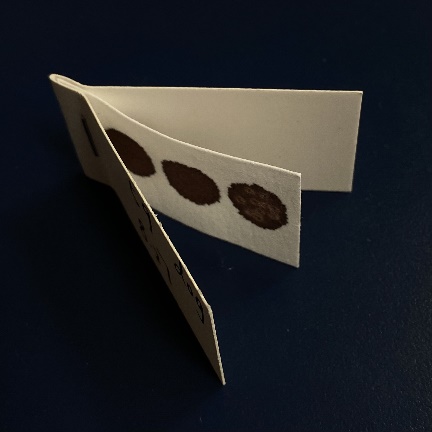
**

**Fig S1.** Self-made blood collection card (CF12)

**Table S2. Primers used to detect pathogens**

| **Parasites** | **Primers** | **Sequences (5'-3')** | **Length (bp)** | **References** |
| --- | --- | --- | --- | --- |
| ***Plasmodium yoelii*** | *P.y*_18s_F | GGGGATTGGTTTTGACGTTTTTGCG | 134 |  |
|  | *P.y*_18s_R | AAGCATTAAATAAAGCGAATACATCCTTAT |  |  |
| ***Plasmodium spp.*** | rPLU6 | TTAAAATTGTTGCAGTTAAAACG | 1003 | [1][2] |
|  | rPLU5 | CCTGTTGTTGCCTTAAACTTC |  |  |
| ***Plasmodium falciparum*** | rFAL1 | TTAAACTGGTTTGGGAAAACCAAATATATT | 205 |  |
|  | rFAL2 | ACACAATGAACTCAATCATGACTACCCGTC |  |  |
| ***Plasmodium vivax*** | rVIV1 | CGCTTCTAGCTTAATCCACATAACTGATAC | 117 |  |
|  | rVIV2 | ACTTCCAAGCCGAAGCAAAGAAAGTCCTTA |  |  |
| ***Babesia gibsoni*** | Bbs_18s_F | GTCTTGTAATTGGAATGATGGTGAC | 339 | [11] |
|  | Bbs_18s_R | ATGCCCCCAACCGTTCCTATTA |  |  |

**Table S3. Twenty commercially available DNA polymerases**

| **Manufacturers** | **DNA polymerases** | **Item No.** | **Tests** | **Price (USD/Kit)** |
| --- | --- | --- | --- | --- |
| **Beyotime** | Hemo*Taq* | D7241S | 200 | 39.70 |
|  | *Taq* | D7205 | 160 | 4.39 |
|  | Hemo*Taq* HF | D7243S | 200 | 47.85 |
|  | BeyoFusion | D7220 | 80 | 16.95 |
| **Sangon biotech** | Direct PCR Kit | B639289-0050 | 50 | 82.37 |
|  | *Taq* Plus | B500013-0100 | 20 | 3.45 |
| **TAKARA** | Tks Gflex | R060Q | 80 | 75.47 |
|  | *Taq* | R001A | 200 | 18.83 |
|  | MightyAmp | R071Q | 40 | 40.95 |
| **TOYOBO** | r*Taq* | TAP-211 | 100 | 23.54 |
|  | KOD Plus Neo | KOD-401S | 20 | 23.54 |
|  | KOD FX Neo | KFX-201S | 20 | 31.38 |
| **TIANGEN** | Mouse Tissue Direct PCR Kit | KG205 | 50 | 62.76 |
|  | Ultra HiFidelity PCR Kit | KP203 | 80 | 97.28 |
| **FOREVER STAR** | Blood Direct PCR Kit | FS-P6001 | 500 | 18.83 |
| **Thermo Fisher** | Phire Hot Start II | F122S | 200 | 220.76 |
|  | Platinum Direct PCR kit | A44647100 | 100 | 136.97 |
| **NEB** | Hemo klen *Taq* | M0332S | 200 | 186.55 |
|  | Vent | M0254V | 200 | 61.03 |
|  | Q5 | M0491S | 100 | 137.92 |

**Table S4.** The PCR condition for amplification of *Plasmodium* DNA^*^

| **DNA polymerases** | | **Predenaturation** | **denaturation** | **annealing** | **extension** | **cycles** | **Final extension** | **Total Time** |
| --- | --- | --- | --- | --- | --- | --- | --- | --- |
| **Beyotime** | **Hemo*Taq*** | 94°C 5min | 94°C 30s | 55°C 30s | 68°C 3min | 35 | 68°C 10min | 3h 2min |
|  | ***Taq*** | 94°C 3min | 94°C 30s | 50°C 30s | 72°C 1min30s | 30 | 72°C 10min | 1h 53min |
|  | **Hemo*Taq* HF** | 94°C 5min | 94°C 30s | 55°C 30s | 68°C 30s | 30 | 68°C 10min | 1h 16min |
|  | **BeyoFusion** | 92°C 3min | 92°C 30s | 55°C 30s | 68°C 30s | 30 | 68°C 10min | 1h 19min |
| **Sangon biotech** | **Direct PCR Kit** | 98°C 30s | 98°C 5s | 55°C 5s | 72°C 30s | 35 | 72°C 1min | 50min |
|  | ***Taq* Plus** | 95°C 5min | 95°C 30s | 55°C 30s | 72°C 1min30s | 25 | 72°C 10min | 1h 38min |
| **TAKARA** | **Tks Gflex** | 94°C 1min | 98°C 10s | 55°C 15s | 68°C 45s | 30 | 68°C 10min | 1h 8min |
|  | ***Taq*** | 94°C 2min | 98°C 10s | 55°C 30s | 72°C 1min30s | 30 | 72°C 10min | 1h 41min |
|  | **MightyAmp** | 98°C 2min | 98°C 10s | 55°C 15s | 68°C 1min30s | 30 | 68°C 10min | 1h 34min |
| **TOYOBO** | **r*Taq*** | 94°C 2min | 94°C 45s | 55°C 45s | 72°C 1min30s | 35 | 72°C 10min | 2h 23min |
|  | **KOD Plus Neo** | 94°C 2min | 98°C 10s | 55°C 30s | 68°C 45s | 35 | 68°C 10min | 1h 28min |
|  | **KOD FX Neo** | 94°C 2min | 98°C 10s | 55°C 30s | 68°C 45s | 35 | 68°C 10min | 1h 28min |
| **TIANGEN** | **Mouse Tissue Direct PCR** | 95°C 3min | 94°C 30s | 55°C 30s | 72°C 1min15s | 35 | 72°C 5min | 2h 42min |
|  | **Ultra HiFidelity PCR** | 94°C 2min | 98°C 10s | 55°C 30s | 68°C 45s | 35 | 68°C 5min | 1h 23s |
| **FOREVER STAR** | **Blood Direct PCR** | 95°C 8min | 95°C 25s | 55°C 25s | 72°C 1min30s | 30 | 72°C 3min | 1h 44min |
| **ThermoFisher** | **Phire Hot Start II** | 98°C 30s | 98°C 5s | 55°C 5s | 72°C 20s | 30 | 72°C 10min | 48min |
|  | **Platinum Direct PCR** | 94°C 2min | 94°C 15s | 55°C 15s | 68°C 30s | 35 | 68°C 10min | 1h 11min |
| **NEB** | **Hemo klen *Taq*** | 95°C 3min | 95°C 20s | 55°C 30s | 68°C 3min | 35 | 68°C 10min | 2h 55min |
|  | **Vent** | 95°C 5min | 95°C 30s | 55°C 30s | 72°C 1min30s | 30 | 72°C 5min | 1h 48min |
|  | **Q5** | 98°C 30s | 98°C 10s | 55°C 30s | 72°C 45s | 30 | 72°C 2min | 1h 9min |

^*^The primers rPLU5/6 are specific to the *Plasmodium* genus, the target fragment length is about 1003 bp.

**Table S5.** The PCR conditions of Eight DNA polymerases for detection of *P. falciparum*^*^

| **DNA polymerases** | **Extension rate** | **Predenaturation** | **denaturation** | **annealing** | **extension** | **Cycles** | **Final extension** | **Total Time** |
| --- | --- | --- | --- | --- | --- | --- | --- | --- |
| **TaKaRa *Taq*** | 1min/kb | 94°C 2min | 98°C 10s | 55°C 30s | 72°C 30s | 30 | 72°C 10min | 1h 10min |
| **Beyotime Hemo*Taq*** | 2min/kb | 94°C 5min | 94°C 30s | 55°C 30s | 68°C 1min | 35 | 68°C 10min | 1h 50min |
| **NEB Hemo klen *Taq*** | 2min/kb | 95°C 3min | 95°C 20s | 55°C 30s | 68°C 1min | 35 | 68°C 10min | 1h 43min |
| **TaKaRa MightyAmp** | 1min/kb | 98°C 2min | 98°C 10s | 55°C 15s | 68°C 30s | 30 | 68°C 10min | 1h 3min |
| **TOYOBO KOD FX Neo** | 30s/kb | 94°C 2min | 98°C 10s | 55°C 30s | 68°C 15s | 35 | 68°C 10min | 1h 12min |
| **Sangon Direct PCR Kit** | 20s/kb | 98°C 30s | 98°C 5s | 55°C 5s | 72°C 10s | 35 | 72°C 1min | 38min |
| **TaKaRa Tks Gflex** | 30s/kb | 94°C 1min | 98°C 10s | 55°C 15s | 68°C 15s | 30 | 68°C 10min | 56min |
| **TIANGEN Ultra HiFidelity PCR Kit** | 30s/kb | 94°C 2min | 98°C 10s | 55°C 30s | 68°C 15s | 35 | 68°C 5min | 1h 5min |

^*^The primers rFAL1/2 are specific to *P. falciparum*, the target fragment length is approximately 205 bp.

***Table S6.*** *Nest PCR conditions of Five cost-effective DNA polymerases for P. falciparum and P. vivax^*^*

|  | **DNA polymerases** | **Predenaturation** | **denaturation** | **annealing** | **extension** | **Cycles** | **Final extension** | **Total Time** |
| --- | --- | --- | --- | --- | --- | --- | --- | --- |
| **Round 1**  **Primers: rPLU5/6** | Beyotime Hemo*Taq* | 94°C 5min | 94°C 30s | 55°C 30s | 68°C 3min | 30 | 68°C 10min | 2h 39min |
|  | NEB Hemo klen *Taq* | 95°C 3min | 95°C 20s | 55°C 30s | 68°C 3min | 30 | 68°C 10min | 2h 33min |
|  | TaKaRa MightyAmp | 98°C 2min | 98°C 10s | 55°C 15s | 68°C 1min30s | 30 | 68°C 10min | 1h 34min |
|  | TOYOBO KOD FX Neo | 94°C 2min | 98°C 10s | 55°C 30s | 68°C 45s | 30 | 68°C 10min | 1h 18min |
|  | Sangon Direct PCR Kit | 98°C 30s | 98°C 5s | 55°C 5s | 72°C 25s | 30 | 72°C 1min | 41min |
| **Round 2**  **Primers: rFLA1/2**  **or rVIV1/2** | Beyotime Hemo*Taq* | 94°C 5min | 94°C 30s | 55°C 30s | 68°C 1min | 35 | 68°C 10min | 1h 50min |
|  | NEB Hemo klen *Taq* | 95°C 3min | 95°C 20s | 55°C 30s | 68°C 1min | 35 | 68°C 10min | 1h 43min |
|  | TaKaRa MightyAmp | 98°C 2min | 98°C 10s | 55°C 15s | 68°C 30s | 35 | 68°C 10min | 1h 10min |
|  | TOYOBO KOD FX Neo | 94°C 2min | 98°C 10s | 55°C 30s | 68°C 15s | 35 | 68°C 10min | 1h 11min |
|  | Sangon Direct PCR Kit | 98°C 30s | 98°C 5s | 55°C 5s | 72°C 10s | 35 | 72°C 1min | 38min |

^*^The primer sets rFAL1/2 and rVIV1/2 are specific to *P. vivax* (about 117 bp) *and P. falciparum* (about 205 bp) respectively.

**Table S7.** The positive rates of eight DNA polymerases for detection of *P. falciparum*

| **DNA polymerases** | **CF12 Filter paper**  **(parasites/µL)** | | | | **Positive rates**  **(n/4)** |
| --- | --- | --- | --- | --- | --- |
|  | **1** | **10^1^** | **10^2^** | **10^3^** |  |
| **TAKARA *Taq*** | - | - | - | ＋ | 25.00% (1/4) |
| **Beyotime Hemo*Taq*** | ＋ | ＋ | ＋ | ＋ | 100.00% (4/4) |
| **NEB Hemo klen *Taq*** | ＋ | ＋ | ＋ | ＋ | 100.00% (4/4) |
| **TAKARA MightyAmp** | ＋ | ＋ | ＋ | ＋ | 100.00% (4/4) |
| **TOYOBO KOD FX Neo** | ＋ | ＋ | ＋ | ＋ | 100.00% (4/4) |
| **Sangon Direct PCR Kit** | ＋ | ＋ | ＋ | ＋ | 100.00% (4/4) |
| **TAKARA Tks Gflex** | - | - | - | ＋ | 25.00% (1/4) |
| **TIANGEN Ultra HiFidelity PCR Kit** | - | - | - | ＋ | 25.00% (1/4) |

**Table S8. The results of five cost-effective DNA polymerases for detection of clinical human malaria DBS samples**

| ***Plasmodium*** | **No.** | **Countries or regions** | **Date** | **DNA polymerases** | | | | |
| --- | --- | --- | --- | --- | --- | --- | --- | --- |
|  |  |  |  | **Beyotime**  **Hemo*Taq*** | **NEB HemoKlen *Taq*** | **TAKARA MightyAmp** | **TOYOBO**  **KOD FX Neo** | **Sangon**  **Direct PCR Kit** |
| ***P. f*** | 1 | Myanmar | 2013.11.10 | ＋ | ＋ | ＋ | ＋ | ＋ |
|  | 2 | Nigeria | 2013.11.28 | **-** | **-** | ＋ | ＋ | ＋ |
|  | 3 | Nigeria | 2014.06.23 | ＋ | ＋ | ＋ | ＋ | ＋ |
|  | 4 | Nigeria | 2014.06.27 | ＋ | ＋ | ＋ | ＋ | ＋ |
|  | 5 | Angola | 2014.06.27 | ＋ | ＋ | ＋ | ＋ | ＋ |
|  | 6 | Benin | 2014.06.30 | ＋ | ＋ | ＋ | ＋ | ＋ |
|  | 7 | Liberia | 2014.07.08 | ＋ | ＋ | ＋ | ＋ | ＋ |
|  | 8 | Nigeria | 2014.07.24 | **-** | **-** | ＋ | ＋ | ＋ |
|  | 9 | Ghana | 2014.08.25 | **-** | **-** | ＋ | ＋ | ＋ |
|  | 10 | Burkina Faso | 2014.08.27 | ＋ | ＋ | ＋ | ＋ | ＋ |
|  | 11 | Liberia | 2014.09.16 | **-** | ＋ | ＋ | ＋ | ＋ |
|  | 12 | Liberia | 2014.09.17 | ＋ | ＋ | ＋ | ＋ | ＋ |
|  | 13 | Congo-Kinshasa | 2014.09.17 | ＋ | ＋ | ＋ | ＋ | ＋ |
|  | 14 | Equatorial Guinea | 2014.09.20 | ＋ | ＋ | ＋ | ＋ | ＋ |
|  | 15 | Uganda | 2014.09.25 | ＋ | ＋ | ＋ | ＋ | ＋ |
|  | 16 | Nigeria | 2014.09.29 | ＋ | ＋ | ＋ | ＋ | ＋ |
|  | 17 | Benin | 2014.10.06 | ＋ | ＋ | ＋ | ＋ | ＋ |
|  | 18 | Nigeria | 2014.11.20 | ＋ | ＋ | ＋ | ＋ | ＋ |
|  | 19 | Rwanda | 2014.11.27 | ＋ | ＋ | ＋ | ＋ | ＋ |
|  | 20 | Congo-Kinshasa | 2014.12.17 | ＋ | ＋ | ＋ | ＋ | ＋ |
|  | 21 | Equatorial Guinea | 2014.12.18 | ＋ | ＋ | ＋ | ＋ | ＋ |
| ***P. v*** | 1 | Wuhe County, Anhui Province, China | 2009.08.26 | ＋ | ＋ | ＋ | ＋ | ＋ |
|  | 2 |  | 2009.08.29 | ＋ | ＋ | ＋ | ＋ | ＋ |
|  | 3 |  | 2009.08.31 | ＋ | ＋ | ＋ | ＋ | ＋ |
|  | 4 |  | 2009.09.07 | ＋ | ＋ | ＋ | ＋ | ＋ |
|  | 5 |  | 2009.09.09 | ＋ | ＋ | ＋ | ＋ | ＋ |
|  | 6 |  | 2009.09.10 | ＋ | ＋ | ＋ | ＋ | ＋ |
|  | 7 |  | 2009.08.08 | ＋ | ＋ | ＋ | ＋ | ＋ |
|  | 8 |  | 2009.08.10 | ＋ | ＋ | ＋ | ＋ | ＋ |
|  | 9 |  | 2009.08.17 | ＋ | ＋ | ＋ | ＋ | ＋ |
|  | 10 |  | 2009.08.17 | ＋ | ＋ | ＋ | ＋ | ＋ |
|  | 11 |  | 2009.08.06 | ＋ | ＋ | ＋ | ＋ | ＋ |
|  | 12 |  | 2009.08.05 | ＋ | ＋ | ＋ | ＋ | ＋ |
|  | 13 |  | 2009.07.28 | ＋ | ＋ | ＋ | ＋ | ＋ |
|  | 14 |  | 2009.08.03 | ＋ | ＋ | ＋ | ＋ | ＋ |
|  | 15 |  | 2014.07.17 | ＋ | ＋ | ＋ | ＋ | ＋ |
|  | 16 |  | 2014.07.10 | ＋ | ＋ | ＋ | ＋ | ＋ |
|  | 17 |  | 2009.08.17 | ＋ | ＋ | ＋ | ＋ | ＋ |
|  | 18 |  | 2009.09.15 | ＋ | ＋ | ＋ | ＋ | ＋ |
|  | 19 |  | 2009.10.19 | ＋ | ＋ | ＋ | ＋ | ＋ |
|  | 20 |  | 2009.08.13 | ＋ | ＋ | ＋ | ＋ | ＋ |
| Positive rates of *P. falciparum* (n/21) | | | | 80.95% (17/21) | 85.71% (18/21) | 100.00% (21/21) | 100.00% (21/21) | 100.00% (21/21) |
| Positive rates of *P. vivax* (n/20) | | | | 100.00% (20/20) | 100.00% (20/20) | 100.00% (20/20) | 100.00% (20/20) | 100.00% (20/20) |
| Total positive rates (n/41) | | | | 90.24% (37/41) | 92.68% (38/41) | 100.00% (41/41) | 100.00% (41/41) | 100.00% (41/41) |

**Table S9. The positive rates of MightyAMP (TAKARA) for detection of animal DBS samples**

| **Cities** | **Dog DBS samples (n)** | **Positive samples (n)** | **Positive rates** |
| --- | --- | --- | --- |
| **Changsha** | 36 | 0 | 0.00% |
| **Bengbu** | 55 | 2 | 3.64% |
| **Nanjing** | 48  (63*) | 0  (15*) | 0.00%  (23.81%*) |

^*^ There were 15 positive samples (single-blinded controls) inserted into clinical samples. The results showed direct PCR could detect *Babesia gibsoni* at 100% positive rate (15/15).
